# System design and habitat type drive microbial communities in recirculating aquaculture systems: comparison of conventional fish-only and sustainable aquaponic systems

**DOI:** 10.3389/fmicb.2026.1706522

**Published:** 2026-02-24

**Authors:** Alberto Ruiz, Karl B. Andree, Dolors Furones, Daniel Scicchitano, Marco Candela, Ricard Carbó, Enric Gisbert

**Affiliations:** 1 Aquaculture Program, Institut de Recerca i Tecnologia Agroalimentaries (IRTA), Ràpita, Spain; 2 Aquaculture and Fisheries Group, Wageningen University and Research, Wageningen, Netherlands; 3 Unit of Microbiome Science and Biotechnology, Department of Pharmacy and Biotechnology, University of Bologna, Bologna, Italy; 4 Fano Marine Center, The Inter-Institute Center for Research on Marine Biodiversity, Resources and Biotechnologies, Pesaro Urbino, Italy

**Keywords:** Biofilm, biological filter, fish microbiota, *Mugil cephalus*, recirculating aquaculture system, *Salicornia patula*, saltwater aquaponics, sustainable food production

## Abstract

This study compares how system design and experimental conditions shape bacterial communities across distinct habitats in a coupled seawater aquaponic system and a marine RAS, and explores their functional implications for system efficiency and productivity. Bacterial communities from fish guts, biofilters, biofilms and water were characterized after 4 months of rearing flathead grey mullet (*Mugil cephalus*) and glasswort (*Salicornia patula*) using 16S rRNA gene sequencing. In the RAS, bacterial richness (Chao1 and ACE) and diversity (Shannon and Simpson) progressively increased across compartments, while they remained stable in the aquaponic system, likely due to the differences in system design such as UV filtration in the RAS. Significant differences in bacterial community structure (weighted UniFrac) and composition were found in the four habitat types compared between systems, reflecting the different design and functionality of each system. In particular, fish gut bacteria were typical teleost commensals associated with positive gut health and disease resistance, dominated by the phylum *Pseudomonadota* and the genus *Pseudomonas* but showing differences in lower abundant taxa between systems. The biofilm and water of the aquaponic system showed genera with plant growth-promoting, disease-resistance and nutrient-cycling properties, at higher abundances than in the RAS (*Mycobacterium, Sulfitobacter, Marivita, Fuerstiella, Blastopirellula, Hoeflea*). Furthermore, the balance of nitrifying (*i.e., Nitrosomonas*) and denitrifying bacteria (*Pseudomonas, Blastopirellula*) in the biofilters of both systems supported efficient nitrogen cycling and water quality maintenance. Collectively, these results demonstrate that microbial assembly in aquaculture systems is governed by system design and habitat type, with potential functional consequences for fish gut health, plant growth, and overall system efficiency, highlighting the promise of integrated marine systems as sustainable food production strategies.

## 1 Introduction

The environmental impact of the agrifood industry, the escalating degradation of agricultural land and the scarcity of natural resources have resulted in a compelling need to explore sustainable and environmentally friendly strategies toward circular food production models (De Korte et al., 2024). Under this context, aquaponics has emerged as a pioneering food production system, combining aquaculture and hydroponics (the soilless cultivation of plants) to create an optimal environment where fish and plants can thrive. This production model is based on the circulation of nutrient-rich water from fish tanks to serve as a non-chemical fertilizer for hydroponics, as well as on microbial processes like nitrification and denitrification that allow the water to be reused (Tyson et al., 2011; Rakocy et al., 2006). This approach offers numerous advantages over more conventional food production systems, including a higher water conservation, efficient nutrient use, reduced reliance on synthetic soil fertilizers, lower waste discharge to the environment, and lower infrastructure costs (Tyson et al., 2011; Greenfeld et al., 2019).

Classical aquaponic systems, known as coupled or closed-loop systems, were described more than three decades ago. These systems integrate the aquaculture and hydroponic units into a single loop, ensuring that water quality is maintained for supporting both fish and plants growth (Monsees et al., 2017). Meanwhile, decoupled aquaponic systems allow for better control of species-specific requirements by circulating the water from fish tanks toward the hydroponic tanks without returning (Monsees et al., 2017). Aquaponic systems can be further classified according to the types of plant culture systems that are coupled to the fish culture tanks. There are gravel beds, floating rafts, or nutrient film techniques (NFT) that can be adapted for plant culture. Each system has its own particularities. Among these, NFT has been seen as the least efficient even though it can facilitate providing more oxygen to the roots of the plants (Lennard and Leonard, 2006; Liang and Chien, 2013). The gravel bed system has the advantage of not requiring a separate biofilter since the gravel media provides increased surface area for more efficient removal of nitrogen; while the floating raft system has the benefit of providing ample room for root growth although at a cost to root aeration in the absence of supplementary air infusion (Okomoda et al., 2022). The overall design of all coupled aquaponic systems closely resembles that of recirculating aquaculture systems (RAS), but with the addition of a hydroponic tank and the optional elimination of the biofilter and solid removal component (Rakocy et al., 2006). Regardless of this similarity, the functional complexity of these systems substantially differs since aquaponic units must ensure the transformation of toxic nitrogen compounds derived from fish metabolism while maximizing the availability of nutrients for plant growth, the latter being in contrast to RAS models (Yavuzcan Yildiz et al., 2017; Preena et al., 2021).

Among the different farmed fish species, flathead grey mullet (*Mugil cephalus*) is gaining attention for its suitability in aquaponic systems with the aim of promoting aquaculture diversification and sustainability (Rossi et al., 2021). This species is widely appreciated in both developing and developed countries for its rapid growth, good adaptability to captivity, for its flesh characteristics and high-value salted cured roe, known as “bottarga” (Khemis et al., 2019). The omnivorous and detritivorous feeding habits of flathead grey mullet and the lower dietary protein requirements of this species in comparison to strictly carnivorous fish have placed this species in the focus for aquaculture diversification (Bertini et al., 2023). In addition, its euryhaline and eurythermal nature makes it an ideal candidate to be reared in a wide range of environmental conditions, including both marine and freshwater systems and in combination with a variety of plant species, as well as under future climate change scenarios (Crosetti, 2016; Rossi et al., 2021; Gisbert et al., 2024).

The success of aquaponic systems relies on the effective control of microbial activities, which are crucial for the overall health of both fish and plants and for ensuring food safety (Kasozi et al., 2021). Nitrification is a crucial process, which takes place through the oxidation of the ammonia excreted from fish and from the microbial degradation of fish feces into nitrite and/or nitrate, the latter being less toxic for the fish. Moreover, the microbial communities provide essential micro- and macro-nutrients to ensure optimal plant growth and improve the water quality (Kushwaha et al., 2023). Bacterial communities in RAS are mainly of heterotrophic members which contribute to the processing of organic wastes, but they also contain chemoautotrophic members, which are involved in ammonia, nitrite, and nitrate conversions (Rurangwa and Verdegem, 2015; Preena et al., 2021). Regarding aquaponic systems, it has been shown that the bacterial communities in each compartment (*i.e*., fish tank, biofilter, hydroponic tank, sump) are also quite different among them, with specialized autotrophic and heterotrophic bacterial groups in each niche associated to specific selection regimes in the system (Schmautz et al., 2017; Kasozi et al., 2021). Nonetheless, a previous study demonstrated that even though the compartments of a freshwater aquaponic system showed a very distinct diversity and composition between them, the bacterial communities exhibited a significant similarity depending on the type of habitat analyzed (*i.e*., water, fish gut, biofilms adhered to the system) regardless of the compartment (Ruiz et al., 2023). The fact that microbial communities are more influenced by the habitat type than by the specific conditions within each compartment of the aquaponic system may indicate the important role of the water as a continuous medium capable of transporting bacteria from biofilms and the microbiota of cultured fish species between compartments (Moschos et al., 2022).

In this context, this study compares the bacterial communities of the water, biofilms, and host microbiota across compartments in a coupled seawater aquaponic system and a marine RAS unit, aiming to understand how each system's design and experimental conditions shape the microbial communities in the water and the rest of habitats influenced by the water, and to shed light on the potential implications of bacterial communities of the distinct habitat types on overall systems' efficiency. Although previous studies have already compared microbial communities across compartments of aquaculture systems, most focus on a single system or on a limited number of compartments between systems, while the present study offers a comprehensive, multi-habitat comparison across two systems with different designs and conditions for a system-wide assessment of microbial assemblages. Through this comparison, this study also supports the potential of sustainable aquaculture recirculating systems which integrate edible plant production with the rearing of low-trophic species and can be designed to operate efficiently without thermoregulation or high operational costs, as an alternative to conventional condition-regulated RAS. Thus, flathead grey mullet was used as a fish model, and the halophyte glasswort (*Salicornia patula*) was integrated in the coupled aquaponic system due to its culinary, nutritional, medicinal, oilseed, forage crop, and wastewater treatment potential (Gunning, 2016; Sánchez Gavilán et al., 2024).

## 2 Material and methods

### 2.1 Ethics statement

All procedures involving animal and plant manipulation and sampling were carried out according to the Spanish (law 1078 32/2007 and Royal Decree 1201/2015) and European legislation (Directive 2010/63/EU of European Parliament and the Council of the European Union). The experimental protocol was authorized by the Ethical Committee of the Institute of Agrifood Research and Technology and the Generalitat of Catalunya, Direcció General de Polítiques Ambientals i Medi Natural (CEEA 11264/2021).

### 2.2 Design of the RAS and aquaponic units

**Figure 1:**
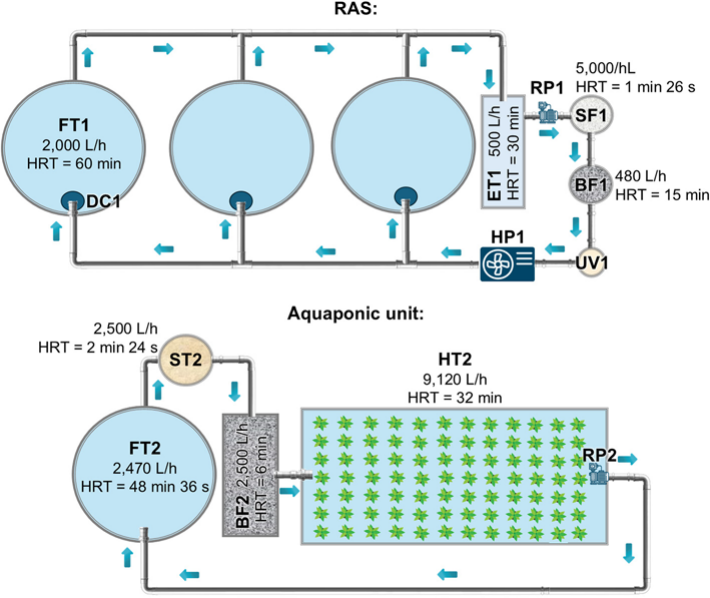
Setup of the recirculating aquaculture system (RAS) unit and aquaponic unit. Components from the RAS: FT1, fish-rearing tanks; ET1, expansion tank; RP1, recirculation pump; SF1, silex sand-filter; BF1, biological filter; UV1, ultraviolet cartridge filter; HP1, heat pump; DC1, degassing column. Components from the aquaponic systems: FT2, fish-rearing tank; ST2, sedimentation tank; BF2, biological filter; HT2, hydroponic tank; RP2, recirculation pump. The hydraulic retention times (HRT) and water flow rates in the different compartments are indicated.

The RAS unit was composed of three independent 2,000 L cylindroconical tanks within an IRTAmar^TM^ water recirculation system (IRTAmar^TM^ BIOTEC, https://irtamar.irta.cat/). The RAS was composed of the following components within this hydraulic circuit (Figure 1): firstly, the saltwater from the fish tanks (FT1) passed to a 250 L expansion tank (ET1), which acted as a water reservoir before it passed to the recirculation pump (RP1). The recirculation pump propelled water (5,000 L/h) toward a silex sand-filter (50 μm, 120 L), where suspended solids (uneaten pellets and fish feces) were retained (SF1). Subsequently, water flowed to a 120 L moving bed biological filter (BF1) filled with High Density Polyethylene (HDPE) bioballs (800 m^2^/m^3^; Acuinuga S.L., Spain) where ammonia was converted into nitrites and nitrates by nitrifying bacteria. Then, the water passed through an ultraviolet cartridge filter (UV1) (HELIOX UV LP, ASTRALPOOL, Spain) equipped with a 48-W UV LP-10 lamp (λ = 254 nm, ASTRALPOOL). After UV1, the water passed to a heat pump (HP1), and subsequently was distributed into three independent degassing columns (50 L; R8) through which it returned to the three fish-rearing tanks. The degassing columns (1 m high) were filled with corrugated plastic which allowed the removal of gases, such as carbon dioxide (CO_2_) and nitrogen (N_2_), due to the mechanical dispersion and mixing of the water, ensuring optimal oxygen levels in the water. As the RAS unit was placed indoors, in a thermoregulated building, air temperature was maintained constant (22.8 ± 0.8). In addition, water temperature (24.1 ± 1.2 C) and dissolved oxygen (5.7 ± 0.1 mg/L) were regulated by an IRTAmar^®^ system. Nevertheless, water temperature and pH (8.1 ± 0.1) were daily measured with an oximeter OXI330 (Crison Instruments, Spain). The levels of ammonia (0.8 ± 0.2 mg NH4^+^/L), nitrite (1.5 ± 0.7 mg ${\text{NO}}_{2}^{-}$/L), and nitrate (3.6 ± 1.6 mg ${\text{NO}}_{3}^{-}$/L) (HACH DR 900 Colorimeter, Hach Company, Spain), and salinity (34.9 ± 0.2‰) (MASTER-20 T Hand-Held Refractometer, ATAGO Co. Ltd., Italy) were measured weekly. Photoperiod was 14 h light and 10 h darkness. The recorded data on temperature, dissolved oxygen, pH, ammonia, nitrite, and nitrate concentrations in the water of the fish tanks from RAS during the experiment are presented in Supplementary Figure 1.

The setup of the aquaponic system was as described in Gisbert et al. (2024) (Figure 1). Three independent aquaponic units were placed within a greenhouse under the same ambient temperatures, photoperiod, and environmental conditions. The aquaponic units operated independently since their hydraulic circuits were not connected. In brief, each aquaponic unit consisted of a 2,000 L cylindroconical tank where fish were kept (FT2). After this, water passed to a sedimentation tank (100 L) where suspended particles were settled and eliminated (ST2), followed by a 125 L biological filter filled with bioballs (500 m^2^/m^3^) (BF2). Then, water flowed by gravity into the hydroponic tank (1.0 x 6.0 x 0.45 m, width, length, and height, respectively; surface area of 6.0 m^2^ and a water volume of 4.8 m^3^) (HT2). *Salicornia patula* (21-23 plants/m^2^) were planted on floating polystyrene rafts. A 0.3 kW air blower ensured strong aeration, providing enough oxygen for the growth of the plant roots and nutrient distribution within the hydroponic tanks. Finally, water was returned (at a flow rate of 2,470 L/h; 0.1 kW) from the hydroponic unit to the fish-rearing tank using a submerged water recirculation pump (RP2). Each aquaponic unit had a total volume of 7.1 m^3^, with a circulation cycle turnover time of approximately 3 h. Both water and air temperatures were ambient, with no provision for thermal regulation in either the aquaponic water or the greenhouse air. Such choices were made to reduce the costs of functioning and maintenance of the aquaponic unit. In the fish tanks, water temperature (24.0 ± 3.1°C), dissolved oxygen (6.3 ± 0.7 mg/L), and pH (7.3 ± 0.4) were daily measured, while the levels of ammonia (0.2-2 mg NH4^+^/L), nitrite (0.2-5 mg ${\text{NO}}_{2}^{-}$/L), and nitrate (0-120 mg ${\text{NO}}_{3}^{-}$/L), and salinity (35.0 ± 0.2‰) were weekly monitored. The photoperiod was the natural one for the date when the experiment was conducted: 13-16 h light and 8-11 h darkness (April-September, latitude 40°37′41′^′^ N). The recorded data on temperature, dissolved oxygen, pH, ammonia, nitrite, and nitrate levels in the water of the fish tanks from the aquaponic units throughout the trial are depicted in Supplementary Figure 2.

Wild flathead grey mullet fry (24.2 ± 0.8 mm standard length, SL; 0.202 ± 0.05 g wet body weight, BW; *N* = 3500) were caught as described by Gisbert and López (2008) and obtained from Pescados y Mariscos Roset S.L. (Deltebre, Spain). The fry were transported to the IRTA facilities in La Ràpita (Tarragona, Spain) and stocked in a 60 m^3^ open-flow raceway, where they were on-grown for experimental purposes using the same commercial feed as in this study (Nutra MP; 1.1 mm pellet size; Skretting, Spain). For the RAS trial, a group of flathead grey mullet juveniles (*n* = 381) with an initial body weight (BW_i_) of 39.4 ± 12.4 g were randomly selected from the raceway and distributed among the three experimental tanks of the RAS unit (127 fish per tank; initial stocking biomass = 2.5 kg/m^3^). For the aquaponic trial, 300 fish from the raceway with BW_i_ = 50.7 ± 12.5 g were distributed in the three aquaponic units (100 fish per tank; initial stocking biomass = 3.5 kg/m^3^). In both RAS and aquaponic systems, the fish were transferred to the rearing tanks just after they were filled, and the same day that *S. patula* was planted in the aquaponic units. One week before stocking the fish and halophytes in both systems, mature bioballs from the same batch with nitrifying bacteria were introduced into both biological filters. Mature bioballs used for filling the biological filters in both RAS and aquaponic units were obtained from a 4,000 L tank with strong aeration in which a suspension of bioballs with nitrifying bacteria were kept with the daily addition of ammonia solution (UN2672) at 25% (v = 25 mL/day), with pH adjustments using bicarbonate as needed. This practice accelerates the maturation of the biological filters in recirculating units and allows standardization of microbial communities in biological filters at the start of trials. The fish were reared in the RAS for 123 days (12^th^ July to 11^th^ November 2021), while the aquaponic trial lasted for 142 days (24^th^ April to 16^th^ September 2021). Flathead grey mullets were fed during both trials with a commercial diet with 55% crude protein, 18% crude fat, and 19.5 MJ/kg digestible energy (Nutra MP; 1.1 mm pellet size; Skretting) with a feeding ration of 2.5% of the stocked biomass using automatic belt feeders. No abnormal behavior, stress, or signs of disease were observed by trained facility personnel throughout the trials.

Wild seedlings of *S. patula* (5.5-7.0 g of initial weight, 8-10 cm of initial length) were collected in April 2021 within the IRTA research facilities, where the species grew naturally around ponds in the Alfacs Bay (40.62778 N, 0.6588874 W; Delta del Ebro, Northeast Spain). Collection was carried out by IRTA staff, as the plants were not taken from external or protected areas. Given the morphologic plasticity of the genus, subsamples were collected for species identification by phylogenetic analysis following the protocols described by Zeinalabedini et al., 2021. For implantation of the salicornia in the aquaponic unit, their roots were cleaned with saltwater to remove the soil attached to them and then planted in the floating rafts of each aquaponic unit (141-145 seedlings per aquaponic unit; initial stocking density = 141-145 g/m^2^). After 2 months of trial, signs of mineral deficiency were detected in the plants; in particular, they started to turn yellow, and their growth was stagnated. Thus, a foliar fertilizer containing a mixture of iron (50,000 ppm), manganese (29,000 ppm), zinc (9,000 ppm), boron (8,000 ppm), copper (3,000 ppm), and molybdenum (400 ppm) (MICROFOL^®^, Microfol Compounding GmbH, Germany) was applied weekly at 0.1% in water by spraying. After its administration once a week for 3 weeks, plants recovered their green color and grew normally.

### 2.3 Sample collection

At the end of each trial, all fish were netted, anesthetized with buffered tricaine methanesulfonate (MS-222, Sigma-Aldrich, Spain; 100 mg/L) and their individual BW and SL were measured. The SL corresponds to the distance between the tip of the nose or upper jaw and the base of the caudal fin. As both trials were run under different temperature regimens, since water monitoring was only done in the RAS unit, somatic growth was compared by means of the thermal growth coefficient (TGC) (Jobling, 2003): TGC (g/degree/day)= {($\sqrt[{3}]{BWf}\;-\;\sqrt[{3}]{BWi}$)/($\sum_{i}^{t}Ti$)} x 1000; where BW_i_ and BW_f_ are the final and initial BW (g), respectively, after t (days), Ti is the mean daily water temperature and $\overset{t}{\underset{i}{\sum }}Ti$ is the sum of mean daily water temperature over the study duration period of t days or degree-days. For comparative purposes with other similar studies, the specific growth rate (SGR) was calculated as 100 x (ln BW_f_-ln BW_i_)/days. The aquaponic plant yield (APY, kg/m^2^) was also calculated taking into consideration the yield of the aerial part of *S. patula* per m^2^ in each aquaponic unit.

For the comparison of the bacterial communities found among the compartments of both types of systems, samples were collected from the following points listed in Table 1. No biological samples were taken at the beginning of the trial since the objective of the study was mainly focused on the comparative analysis of both systems rather than their evolution over time. In the RAS, five fish per tank (*n* = 15), were euthanized with an overdose of the anesthetic MS-222 (300 mg/L) and eviscerated to collect samples of gut microbiota. To ensure the collection of only the autochthonous microbiota adherent to the gut mucosa, fish were fasted for 48 h before the sampling (Viver et al., 2023). The intestinal tract just after the pyloric caeca to the anus was dissected and aseptically opened lengthwise, and the inner walls were gently but insistently scraped with a round-edge spatula to recover the mucosal content, which was individually placed in 2 mL Eppendorf tubes (FIM1). The same procedure was followed to collect the gut mucosal content of five 48 h fasted-fish per tank (*n* = 15) from the aquaponic unit (FIM2). Following the procedure of previous articles (Wold et al., 2014; Bakke et al., 2017; Lorgen-Ritchie et al., 2021), in the RAS unit, three water samples of a volume of 50 mL were also taken in sterile Falcon tubes, from the fish-rearing tank (one of each tank; WFT1), from the sand-filter (three from the same tank; WSF1), and from the biological filter (three from the same tank; WBF1). Water samples of 50 mL were also collected from the sedimentation tank (WST2), and from the hydroponic tank (BHT2) of each of the three hydroponic units. Each water sample was separately filtered through a sterile cellulose acetate membrane filter with a pore size of 0.22 μm (Ø = 25 mm; ref. 10404106; Millipore, MERK, Spain) and, using sterile tweezers, transferred to 5 mL Eppendorf tubes for storage. Furthermore, three samples of biofilm were taken from the walls of the fish tanks (one of each tank; BFT1), expansion tank (BET1), and one sample of each degassing column of the RAS (*n* = 3; BDC1), and from the walls of the hydroponic tank (one of each aquaponic unit; *n* = 3; BHT2). Samples were taken using sterile cotton swabs (5,100/SG/CS, Aptaca, Canelli, Italy) by repeatedly streaking across different areas of the compartment surface to ensure complete coverage. Then, the head of each swab was cut off with sterile scissors and placed into a 5 mL Eppendorf tube. In addition, a sample of the distal part of the roots (*ca*. 3 cm) from the glassworts (BRG2) was collected from each aquaponic unit. From the biological filter tank of each system, several bioballs were collected and cut into smaller pieces which were stored in 5 mL Eppendorf tubes (*n* = 3 for each system). All above-described samples collected were individually frozen at – 80 °C until DNA extraction.

**Table 1:** Sampling points for microbiota collection from the recirculating aquaculture system (RAS) unit and aquaponic unit.

**RAS unit**		**Aquaponic unit**	
FIM1	Fish intestinal mucosa	FIM2	Fish intestinal mucosa
WFT1	Water from the fish tanks	WST2	Water from the sedimentation tank
BFT1	Biofilm from the walls of the fish tanks	BBF2	Bioballs from the biological filter
BET1	Biofilm from the wall of the expansion tank	WHT2	Water from the hydroponic tanks
WSF1	Water from the sand filter	BHT2	Biofilm from the walls of the hydroponic tanks
WBF1	Water from the biological filter	BRG2	Biofilm from the roots of glassworts
BBF1	Bioballs from the biological filter		
BDC1	Biofilm from degassing columns		

### 2.4 Extraction of DNA, sequencing, and data analysis

Different protocols for DNA extraction were applied depending on the type of habitat (*i.e*., fish gut, water, salicornia roots, biofilm, biological filter) in order to maximize the microbial yield. For gut mucosa (*ca*. 250 mg), the DNeasy Blood & Tissue Kit (QIAGEN, Germany) was used as described by Bertini et al. (2023). The DNA of the filtered water was extracted with the DNeasy PowerWater kit (QIAGEN, Germany), and the DNA of the biofilms collected with the swabs, and present in the bioballs and glasswort roots were extracted using the DNeasy PowerBiofilm kit (QIAGEN, Germany). The concentration and purity of extracted DNA were measured in a NanoDrop ND-1000 spectrophotometer (NanoDrop Technologies, Wilmington, DE). The V3-V4 region of the 16S rRNA bacterial genes was amplified with the 341F 5′-CCTACGGGNGGCWGCAG3′) and 785R 5′-GACTACHVGGGTATCTAATCC3′) primers with Illumina overhang sequencing adapters and 2xKAPA HiFi HotStart ReadyMix (KAPA Biosystems), according to the 16S Metagenomic Sequencing Library Preparation Guide of Illumina (2013). Libraries were cleaned up using Agencourt AMPure XP magnetic beads, indexed using Nextera Technology by limited-cycle PCR reaction and then normalized to 4 nM and pooled. Pooled libraries were denatured with 0.2 N NaOH and diluted to 6 pM with 20% PhiX control. Sequencing was performed on Illumina MiSeq platform (2 × 250 bp paired-end; Illumina, San Diego, CA). Raw sequencing data and metadata for all samples included in this study were uploaded to the Sequence Read Archive (SRA) of NCBI repository, with Bioproject accession number PRJNA1309991, https://www.ncbi.nlm.nih.gov/bioproject/PRJNA1309991.

The forward and reverse primers from the raw sequencing data were removed with the tool *Cutadapt* in QIIME2 (v2022.2) (Beiko et al., 2018). Data analysis was performed with a workflow based on the open-source package DADA2 (v1.24.0) in RStudio (v2023.06.1), which models and corrects errors derived from Illumina sequencing (Callahan et al., 2016). The DADA2 package resolves differences at the single-nucleotide level and the end products are amplicon sequence variants (ASVs). In brief, forward and reverse reads were subjected to quality filtering, establishing an individual and average quality threshold of 28, with truncation lengths of 240 nt and 220 nt for forward and reverse reads, respectively. Reads with an expected error higher than 2 were removed from the analysis. Subsequently, paired-end reads were merged, and sequences with an overlap length of less than 12 nucleotides, more than 0 mismatches, or identified as chimeras were discarded. The resultant ASVs were taxonomically classified according to the SILVA database (v138.1) (Quast et al., 2012). Those ASVs identified as mitochondria and chloroplasts were excluded from the analysis, and ASVs with a bootstrapping confidence below 80% were classified as unassigned (Smith et al., 2020).

### 2.5 Statistical analysis

After verifying normal distribution (Shapiro-Wilk test) and homoscedasticity (Levene's test) of data, a Student's *t*-test was used for testing significant differences in TGC and survival of the fish between both systems (*P* ≤ 0.05) using SigmaPlot (v15; Systat Software Inc., USA). Significant differences in alpha diversity indicators of estimated richness (Chao1 and ACE indices) and diversity (Shannon and Simpson indices) and in the relative abundance of bacterial taxa were calculated using the Kruskal-Wallis test followed by the Wilcoxon *post-hoc* test. A permutational multivariate analysis of variance (PERMANOVA) was performed to check significant differences in beta diversity based on weighted UniFrac distances, followed by pairwise PERMANOVAs for multiple comparison. Considering the low number of methodological replicates of the different types of water and biofilm samples (*n* = 3), the *P* value was set to 0.1 for determination of significance. The statistical analyses of the comparison of the samples of microbiota from both aquaculture systems were conducted in the R package *microeco* (v0.12.0; Liu et al., 2021).

## 3 Results

### 3.1 Fish and plant productive performance in RAS and aquaponic units

Flathead grey mullet juveniles were satisfactorily adapted to both rearing systems tested, aquaponics and RAS, as indicated by the respective survival rates ranging from 98.3 to 99% (Table 2; *P* > 0.05). Regarding growth performance, TGC values from flathead grey mullet reared in the aquaponic units (0.84 ± 0.01 g/°C/day) were significantly higher than those of their congeners from RAS tanks (0.67 ± 0.02 g/°C/day) (*P* < 0.001). Concerning halophytes, the species was identified using nuclear ribosomal DNA sequences as *S. patula*. The plant survival was 97.6 ± 1.5% and APY ranged from 2.1 to 2.3 kg/m^2^.

**Table 2:** Growth performance and survival of flathead grey mullet (*Mugil cephalus*) reared in the recirculatory aquaculture system (RAS) and coupled aquaponic system for 123 and 142 days, respectively.

**Flathead grey mullet**	**RAS**	**Aquaponic system**
BW_i_ (g)	39.4 ± 12.41	50.7 ± 12.52
SL_i_ (cm)	12.6 ± 0.18 ^b^	13.5 ± 0.21 ^a^
BW_f_ (g)	148.3 ± 1.17 ^b^	188.7 ± 5.95 ^a^
SL_f_ (cm)	22.6 ± 2.22	22.8 ± 2.71
SGR (%BW/day)	1.00 ± 0.01	0.99 ± 0.01
TGC (g/°C/day)	0.63 ± 0.01 ^b^	0.84 ± 0.01 ^a^
Survival (%)	99.0 ± 1.0	98.3 ± 1.2
**Salicornia**	**RAS**	**Aquaponic system**
Plant yield (g/plant)	-	740.3 ± 66.89
APY (kg/m^2^)	-	7.2 ± 0.55
Survival (%)	-	96.7 ± 1.52

Different letters indicate the presence of statistically significant differences between experimental groups (*t*-test, *P* < 0.05).

### 3.2 Comparison of bacterial richness and diversity among the different microbial habitats from RAS and aquaponic units

**Figure 2:**
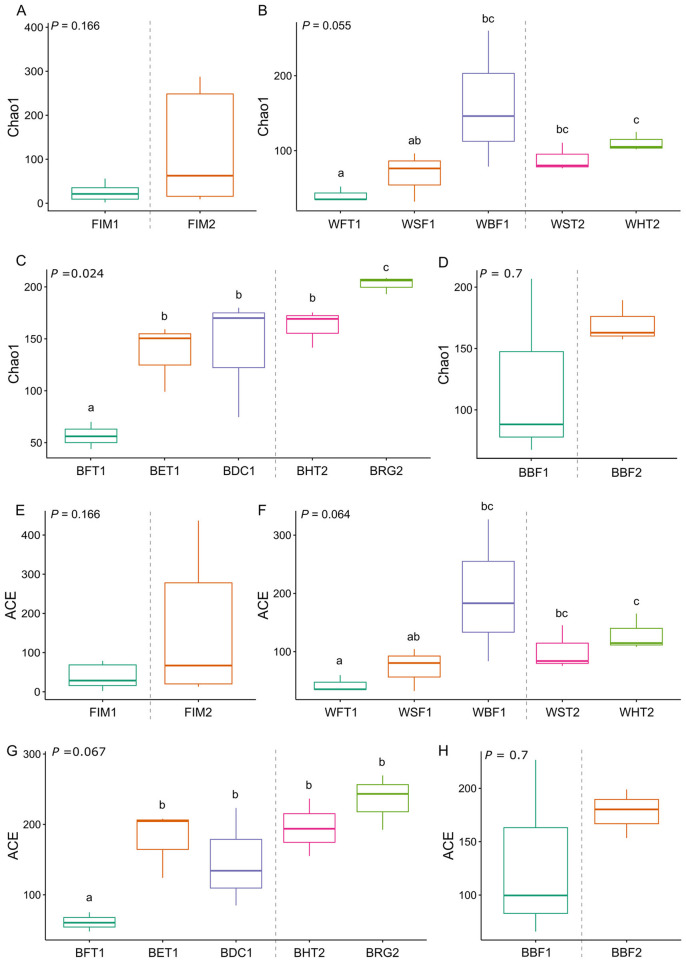
Values of richness for the bacterial communities associated to the compared matrices of the recirculating aquaculture system (RAS) and aquaponic system. Chao1 index for **(A)** fish intestinal microbiota; **(B)** water samples for each compartment of each system; **(C)** biofilm samples from each compartment of each system; **(D)** bioballs from each biofilter unit of each system. ACE index for **(E)** fish intestinal microbiota; **(F)** water samples for each compartment of each system; **(G)** biofilm samples from each compartment of each system; **(H)** bioballs from each biofilter unit of each system. Sampling points from the RAS: FIM1, flathead grey mullet (*Mugil cephalus*) intestinal mucosa; WFT1, water from the fish tanks; BFT1, biofilm from the walls of the fish tanks; BET1, biofilm from the walls of the expansion tank; WSF1, water from the sand filter; WBF1, water from the biological filter; BBF1, bioballs from the biological filter; BDC1, biofilm from the degassing column. Sampling points from the aquaponic systems: FIM2, flathead grey mullet intestinal mucosa; WST2, water from the sedimentation tank; BBF2, bioballs from the biological filter; WHT2, water from the hydroponic tanks; BHT2, biofilm from the walls of the hydroponic tanks; BRG2, biofilm from the roots of glasswort. The different letters represent significant differences among sampling points (Kruskal-Wallis test followed by Wilcoxon test, *P* < 0.1).

Estimated bacterial richness and diversity of the different sampling points from RAS and aquaponic units in terms of ACE and Shannon indices are depicted in Figure 2. Chao1 and ACE indices were similar among the intestines of fish reared in the RAS (FIM1) and aquaponic tanks (FIM2) (Figures 2A, E, respectively). Regarding water, the bacterial richness (Chao1 and ACE indices) in the sedimentation tanks of the aquaponic units (WST2) was significantly higher than in the fish-rearing tanks (WFT1) of the RAS (Wilcoxon test, *P* < 0.1), while it was similar to that of the water collected from the sand (WSF1) and biological filters (WBF1) of the RAS (*P* > 0.1). In the hydroponic tanks, bacterial richness in the water (WHT2), compared with the RAS system, was also significantly higher than in WFT1 and WSF1 (*P* < 0.1), but similar to the water from the biological filter (*P* > 0.1; Figures 2B, F). In addition, the biofilm from the walls of the fish tanks (BFT1) showed lower bacterial richness (Chao1 and ACE indices) than the biofilms from the expansion tank (BET1) and degassing columns (BDC1) of the RAS unit, and than the biofilms from the hydroponic tanks (BHT2) and roots of glasswort (BRG2) (*P* < 0.1; Figures 2C, G). In addition, the Chao1 values were also significantly higher in BRG2 in comparison to BET1, BDC1 and BHT2 (*P* < 0.1; Figure 2C). On the other hand, no significant differences in bacterial richness were found between the bioballs of the biological filters from RAS (BBF1) and aquaponic units (BBF2) (*P* > 0.1) when using Chao1 (Figure 2D) and ACE indices (Figure 2H).

**Figure 3:**
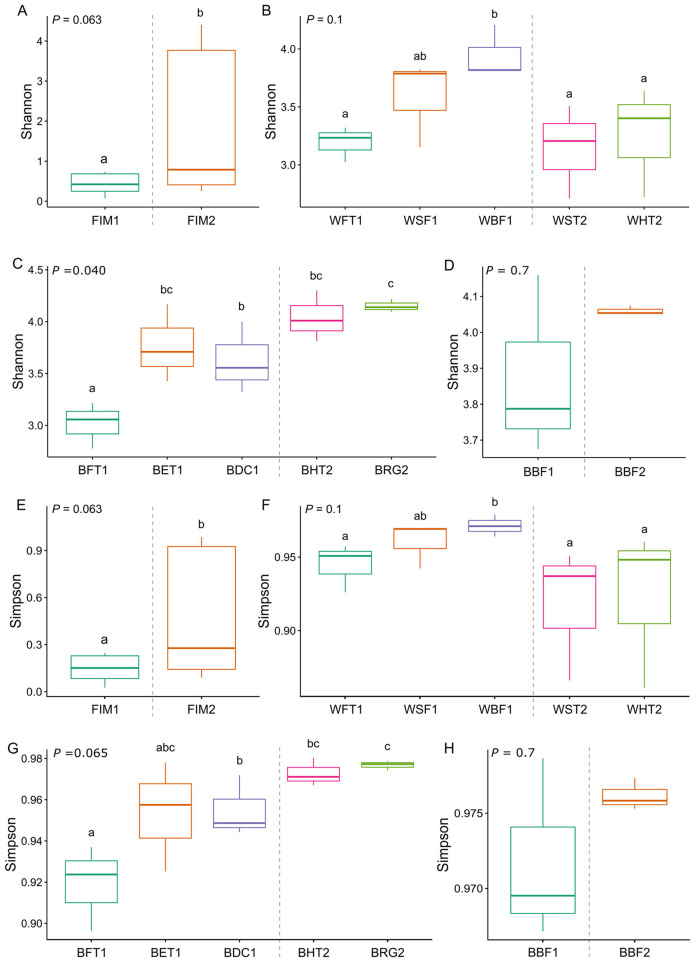
Values of diversity for the bacterial communities associated to the compared matrices of the recirculating aquaculture system (RAS) and aquaponic system. Shannon index for **(A)** fish intestinal microbiota; **(B)** water samples for each compartment of each system; **(C)** biofilm samples from each compartment of each system; **(D)** bioballs from each biofilter unit of each system. Simpson index for **(E)** fish intestinal microbiota; **(F)** water samples for each compartment of each system; **(G)** biofilm samples from each compartment of each system; **(H)** bioballs from each biofilter unit of each system. Sampling points from the RAS: FIM1, flathead grey mullet (*Mugil cephalus*) intestinal mucosa; WFT1, water from the fish tanks; BFT1, biofilm from the walls of the fish tanks; BET1, biofilm from the walls of the expansion tank; WSF1, water from the sand filter; WBF1, water from the biological filter; BBF1, bioballs from the biological filter; BDC1, biofilm from the degassing column. Sampling points from the aquaponic systems: FIM2, flathead grey mullet intestinal mucosa; WST2, water from the sedimentation tank; BBF2, bioballs from the biological filter; WHT2, water from the hydroponic tanks; BHT2, biofilm from the walls of the hydroponic tanks; BRG2, biofilm from the roots of glasswort. The different letters represent significant differences among sampling points (Kruskal-Wallis test followed by Wilcoxon test, *P* < 0.1).

In terms of diversity, the values of Shannon and Simpson indices were significantly higher in the gut bacterial communities of flathead grey mullet from the aquaponic system than from the RAS (*P* < 0.1; Figures 3A, E, respectively). Water from the sedimentation and hydroponic tanks of the aquaponic system showed a Shannon and Simpson indices similar to those of the water from the fish-rearing tanks and sand-filter of the RAS (*P* > 0.1), and significantly lower than the water of the RAS biological filter (*P* < 0.01; Figures 3B, F). The biofilms from the walls of the hydroponic tanks and roots of glasswort showed a similar Shannon index to that from the biofilm of the RAS expansion tank (*P* > 0.1), whereas it was significantly higher than that from the biofilms of the fish tanks and degassing columns of the RAS (*P* < 0.1; Figure 3C). The same pattern was observed for Simpson diversity index (Figure 3G); however, in this case, Simpson indices were similar between the expansion tank wall biofilms and those from the fish tanks (*P* > 0.01; Figure 3G), whereas Shannon diversity values were higher in the expansion tank wall biofilms than in the fish tanks (*P* < 0.01; Figure 3G). No differences were observed in Shannon nor Simpson index between the biological filters of both compared systems (*P* > 0.1; Figures 3D, H, respectively).

### 3.3 Comparison of bacterial community structure among the different microbial habitats from RAS and aquaponic units

**Figure 4:**
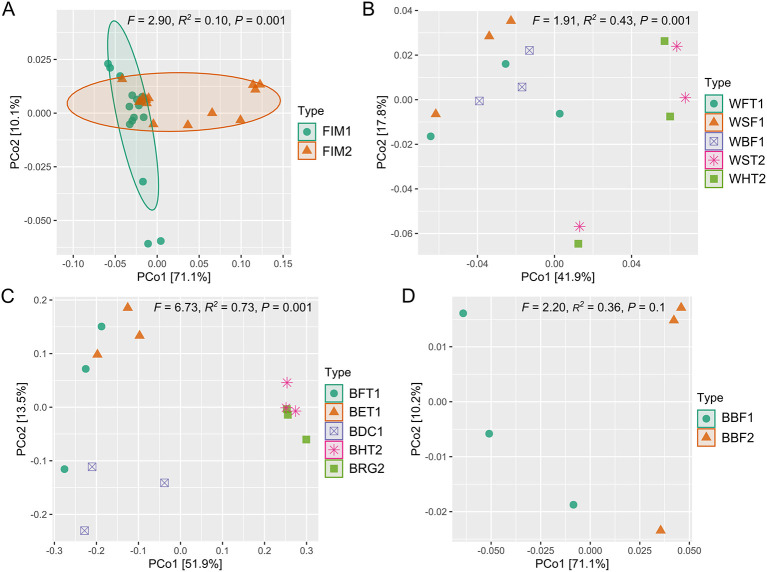
PCoA analyses showing the spatial distribution based on weighted UniFrac distances of the bacterial communities associated to the compared matrices of the recirculating aquaculture system (RAS) and aquaponic system. **(A)** Fish intestinal microbiota; **(B)** Water samples for each compartment of each system; **(C)** Biofilm samples from each compartment of each system; **(D)** Bioballs from each biofilter unit of each system. Sampling points from the RAS: FIM1, flathead grey mullet (*Mugil cephalus*) intestinal mucosa; WFT1, water from the fish tanks; BFT1, biofilm from the walls of the fish tanks; BET1, biofilm from the walls of the expansion tank; WSF1, water from the sand filter; WBF1, water from the biological filter; BBF1, bioballs from the biological filter; BDC1, biofilm from the degassing column. Sampling points from the aquaponic systems: FIM2, flathead grey mullet intestinal mucosa; WST2, water from the sedimentation tank; BBF2, bioballs from the biological filter; WHT2, water from the hydroponic tanks; BHT2, biofilm from the walls of the hydroponic tanks; BRG2, biofilm from the roots of glasswort.

In terms of beta diversity, when quantitatively considering the phylogenetic relationships among ASVs (weighted UniFrac distances), there was a clear and significant separation between the bacterial communities found in the intestines of the fish from both systems, as well as between the different types of biofilms and bioballs from both systems (PERMANOVA, *P* < 0.1; Figure 4). On the other hand, the water from both systems also showed significant differences in weighted UniFrac distances between them (Figure 4B), except for the water from the fish tanks compared to the water collected from the sand filter (*F* = 1.15, *R*^2^ = 0.22, *P* = 0.4) and from the biological filter of the RAS system (*F* = 1.50, *R*^2^ = 0.27, *P* = 0.2). Within the aquaponic system, there were no statistically significant differences between the water of the sedimentation tank and the water of the hydroponic tanks (*F* = 0.14, *R*^2^ = 0.03, *P* = 0.7).

### 3.4 Comparison of bacterial composition among the different microbial habitats from RAS and aquaponic units

**Figure 5:**
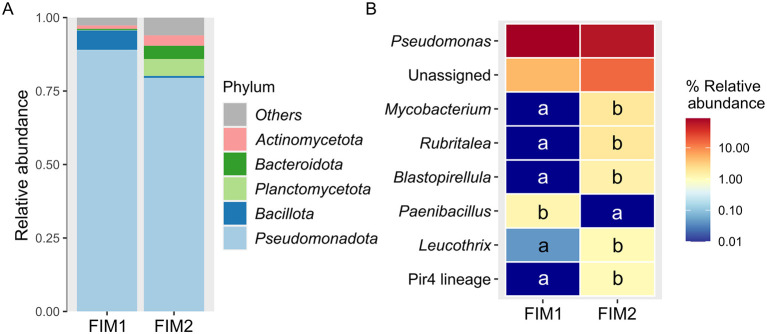
Taxonomic assessment of fish intestinal microbiota. Relative abundance of the bacterial communities at the level of phylum **(A)** and genus **(B)** associated to the intestinal mucosa of flathead grey mullet (*Mugil cephalus*) reared in a recirculating aquaculture system (FIM1) and in an aquaponic unit (FIM2). The phyla with a relative abundance < 2% were classified as Others, whereas genera with a relative abundance lower than 0.5% were not depicted. The different letters in the heatmap represent the existence of significant differences in relative abundance among genera (Kruskal-Wallis test, *P* < 0.1).

The composition of the intestinal mucosa of flathead grey mullet in terms of relative abundance at the level of phylum and genus is represented in Figures 5A, B, respectively. The relative abundance of the phylum *Bacillota* (0.66 ± 0.29%, mean ± SEM) in the gut of fish from the aquaponic unit (FIM2) was significantly lower than in their congeners from the RAS (FIM1, 6.58 ± 2.55; Kruskal-Wallis test, *P* < 0.1), whereas *Planctomycetota, Bacteroidota* and *Actinomycetota* phyla showed a significant higher relative abundance in FIM2 (5.77 ± 1.75, 4.49 ± 1.82, and 3.55 ± 0.99%) than in FIM1 (0.18 ± 0.14, 0.35 ± 0.21, and 1.15 ± 0.80, respectively; *P* < 0.1). At the genus level, the intestines of all fish were dominated by *Pseudomonas* (87.02 ± 3.71% in FIM2; 62.25 ± 10.28% in FIM1). In addition, in the intestinal mucosa of fish from the aquaponic system there were found many genera that were absent in the fish from the RAS, such as *Mycobacterium* (1.80 ± 0.67%), *Rubritalea* (1.75 ± 0.66%) *Blastopirellula* (1.37 ± 0.54%), *Pirellula*-related Pir4 lineage (1.04 ± 0.33) and *Leucothrix* (1.09 ± 0.54%), whose abundance in the fish from the RAS was nearly absent (0.04 ± 0.04%; *P* < 0.1).

**Figure 6:**
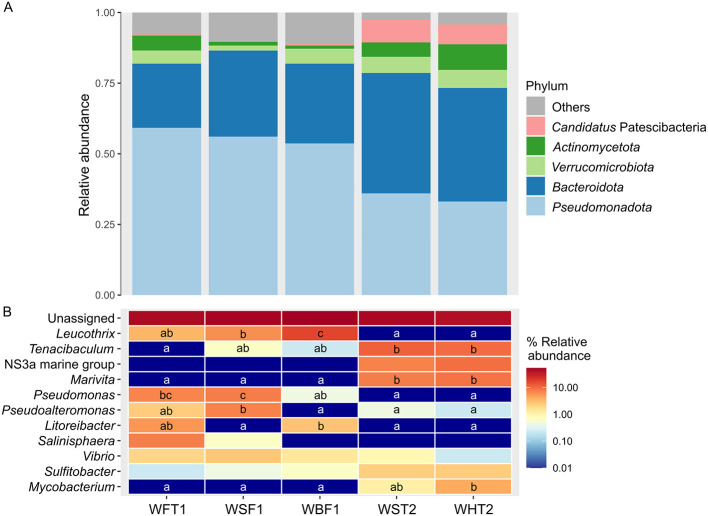
Taxonomic assessment of water samples. Relative abundance of the bacterial communities at the level of phylum **(A)** and genus **(B)** associated to water samples collected at different points within the hydraulic circuit of a recirculating aquaculture system (RAS) and aquaponic units. Sampling points from the RAS: WFT1, water from the fish tanks; WSF1, water from the sand filter; WBF1, water from the biological filter. Sampling points from the aquaponic systems: WST2, water from the sedimentation tank; WHT2, water from the hydroponic tanks. The phyla with a relative abundance < 2% were classified as Others, whereas genera with a relative abundance lower than 1% were not depicted. The different letters in the heatmap represent the significant differences among genera (Kruskal-Wallis test followed by Wilcoxon test, *P* < 0.1).

Concerning water, at both levels of phylum and genus no differences were found between the two sampled points within the aquaponic systems, the sedimentation (WST2) and the hydroponic tanks (WHT2) (*P* > 0.1; Figures 6A, B). Compared to the compartments of the RAS, the relative abundance of *Pseudomonadota* was significantly lower in WST2 (35.98 ± 4.63%) and WHT2 (33.11 ± 5.94%) than in the water from the fish tanks (WFT1, 59.16 ± 5.22%), sand filter (WSF1, 56.07 ± 5.08%) and biological filter (WBF1, 53.64 ± 4.05%) within the RAS unit (*P* < 0.1). Inversely, the levels of *Candidatus* Patescibacteria were significantly higher in WST2 (8.17 ± 5.57%) and WHT2 (7.06 ± 3.92%) than in WFT1 (0.44 ± 0.44%), WSF1 (0.22 ± 0.22%), and WBF1 (0.66 ± 0.00%) (*P* < 0.1). At the level of genus, compared to WFT1, the relative abundance of *Pseudomonas* in the water of the aquaponic system (0.00 ± 0.00% in WST2 and WHT2) was lower compared to the RAS unit (7.06 ± 3.25% in WFT1). Conversely, *Tenacibaculum* and *Marivita*, which were absent in WFT1, were found at high abundances in WST2 (11.48 ± 8.02 and 7.73 ± 3.55%, respectively) and in WHT2 (9.49 ± 6.94 and 8.61 ± 4.51%, respectively). Similarly, an absence of *Marivita* was found in WSF1 and WBF1, while the relative abundances of *Leucothrix*, which was absent in WST2 and WHT2, were 6.18 ± 0.80% in WSF1 and 17.00 ± 2.11 % in WBF1. In addition, WSF1 showed a high abundance of *Pseudomonas* (7.73 ± 4.48%) and *Pseudoalteromonas* (7.28 ± 2.33%), which were near to zero abundance in the water of the aquaponic system. Additionally, WHT2 showed a relative abundance of *Mycobaterium* of 4.19 ± 1.92%, whereas this genus was absent in the water of the RAS unit.

**Figure 7:**
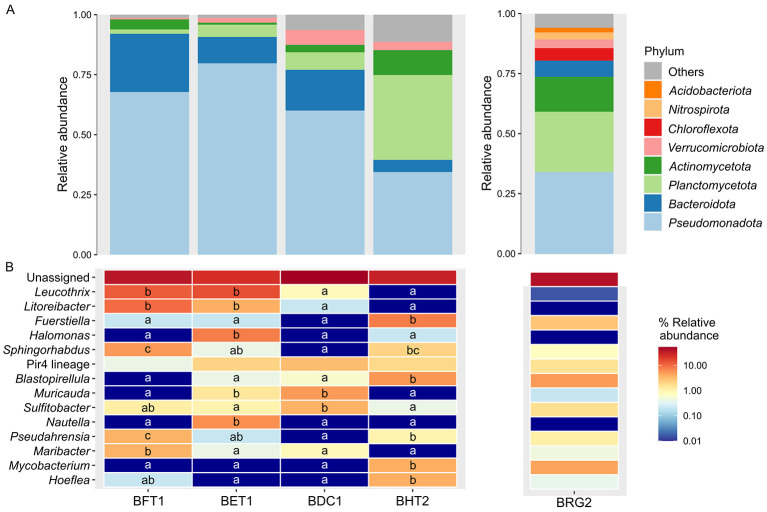
Taxonomic assessment of biofilm samples. Relative abundance of the bacterial communities at the level of phylum **(A)** and genus **(B)** associated to the biofilm samples collected at different points within the hydraulic circuit of a recirculating aquaculture system (RAS) and aquaponic units. Sampling points from the RAS: BFT1, biofilm from the walls of the fish tanks; BET1, biofilm from the walls of the expansion tank; BDC1, biofilm from the degassing column. Sampling points from the aquaponic systems: BHT2, biofilm from the walls of the hydroponic tanks; BRG2, biofilm from the roots of glasswort. The phyla with a relative abundance < 2% were classified as Others and the genera < 1% were not depicted. The different letters in the heatmap represent the significant differences among genera (Kruskal-Wallis test followed by Wilcoxon test, *P* < 0.1). The relative abundance taxa of the biofilm samples from abiotic (swabbed samples) and biotic (roots) surfaces was not statistically compared due to the distinct composition found in the two types of matrices.

Regarding the biofilm samples, at the level of phylum the relative abundance of *Pseudomonadota* (34.44 ± 3.13%), and *Bacteroidota* (5.08 ± 0.80%) in the biofilms from the hydroponic tanks (BHT2) were significantly lower than the biofilms collected in different locations within the RAS system like from the walls of the fish tanks (BFT1, 67.77 ± 3.87 and 24.28 ± 2.49%), the expansion tank (BET1, 79.69 ± 5.85 and 11.04 ± 1.10%) and the degassing columns (BDC1, 60.04 ± 5.16 and 17.00 ± 3.91%) (*P* < 0.1; Figure 7A). On the other hand, the levels of *Planctomycetota* (35.32 ± 1.34%) and *Actinomycetota* (10.38 ± 3.47%) were significantly higher than in BFT1 (1.77 ± 0.58 and 4.19 ± 2.21%), BET1 (5.08 ± 2.17 and 0.88 ± 0.58%), and BDC1 (7.28 ± 1.75 and 3.09 ± 0.22%) (*P* < 0.1). *Pseudomonadota, Planctomycetota, Actinomycetota*, and *Bacteroidota* were also highly abundant in the roots of glasswort (WBF1, 34.18 ± 3.83, 25.05 ± 3.25, 14.6 ± 3.09, and 6.51 ± 0.48%, respectively).

At the level of genus, *Leucothrix* (14.57 ± 4.41 and 17.88 ± 10.48%) and *Litoreibacter* (11.48 ± 3.47 and 4.42 ± 1.72%) were highly abundant in BFT1 and BET1, while they were almost absent in the rest of the biofilms from both systems (*P* < 0.1; Figure 7B). Additionally, BFT1 was also dominated by *Sphingorhabdus* (6.18 ± 1.55%), *Pseudahrensia* (4.19 ± 1.34%), and *Maribacter* (4.19 ± 0.96%), while *Halomonas* (9.05 ± 6.76%) and *Nautella* (7.06 ± 4.56%) were the most abundant in BET1. In the case of BDC1, the above-mentioned genera were absent or appeared with relative abundance levels close to zero, while *Muricauda* (5.96 ± 1.99) and *Sulfitobacter* (4.19 ± 1.23) were found at higher abundances that in the rest of the biofilm samples. Regarding BHT2, it was dominated by *Fuerstiella* (9.05 ± 1.55%), *Blastopirellula* (6.62 ± 1.75%), *Mycobacterium* (4.64 ± 3.65%) and *Hoeflea* (4.42 ± 2.55%). In WBF1, the most abundant genera were *Blastopirellula* (7.96 ± 1.63%), *Mycobacterium* (7.87 ± 1.79%), *Bythopirellula* (4.8 ± 0.51%) and *Fuerstiella* (3.36 ± 1.32%).

**Figure 8:**
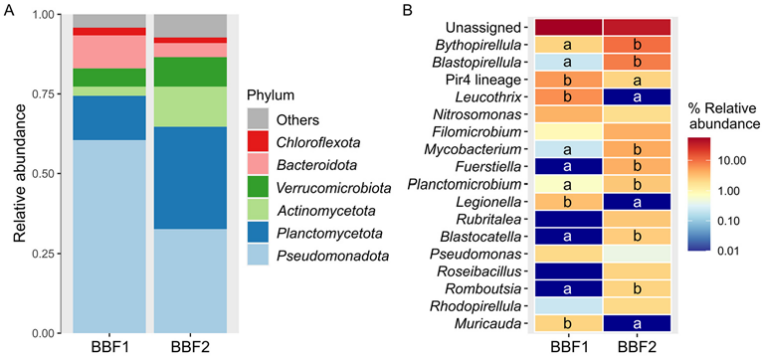
Taxonomic assessment of the bioballs. Relative abundance at the level of phylum **(A)** and genus **(B)** of the bacterial communities associated to the bioballs of the biological filters from the recirculating aquaculture system (BBF1) and of the aquaponic unit (BBF2). The phyla with a relative abundance < 2% were classified as Others and the genera < 1% were not depicted. The different letters in the heatmap represent the significant differences among genera (Kruskal-Wallis test followed by Wilcoxon test, *P* < 0.1).

The composition of the bacterial communities adherent to the bioballs was different between the biological filters of both systems (Figures 8A, B). In the bioballs from the aquaponic system (BBF2), the relative abundance of *Pseudomonadota* (32.67 ± 1.55%) and *Bacteroidota* (4.42 ± 0.80%) was lower than in the RAS unit (BBF1, 60.49 ± 9.58% and 10.38 ± 2.57%, respectively). Inversely, the relative abundance of *Planctomycetota* (32.01 ± 2.87%) and *Actinomycetota* (12.58 ± 1.01%) was higher in BBF2 than in BBF1 (13.91 ± 6.10% and 2.87 ± 3.19%). The bioballs from the aquaponic system were mainly dominated by *Bythopirellula* (9.93 ± 1.38%), *Blastopirellula* (8.17 ± 1.77%), *Mycobacterium* (4.42 ± 1.34%), *Filomicrobium* (3.97 ± 1.01%), and *Fuerstiella* (3.97 ± 2.33%), while the bioballs of the RAS were dominated by *Leucothrix* (6.40 ± 0.44%), the *Pirellula*-related Pir4 lineage (5.52 ± 0.22%), *Nitrosomonas* (3.75 ± 0.44%; *P* > 0.1), and *Legionella* (3.09 ± 0.96%). In addition, there were also significant differences in the abundance of *Planctomicrobium* (0.66 ± 0.38% in BBF1 *vs*. 2.65 ± 0.38% in BBF2), *Blastocatella* (0.00 ± 0.00% in BBF1 *vs*. 2.43 ± 0.88% in BBF2), *Romboutsia* (0.00 ± 0.00% in BBF1 *vs*. 2.21 ± 0.58% in BBF2), and *Muricauda* (2.21 ± 0.22% in BBF1 *vs*. 0.00 ± 0.00% in BBF2).

## 4 Discussion

To effectively evaluate the microbiome composition in different compartments of the hydraulic circuits of both RAS and aquaponic units and extract relevant information about their structure and composition, it was essential to first ensure the performance of both systems in terms of fish biomass production.

### 4.1 Productive performance of RAS and aquaponic systems

The family Mugilidae includes various species that are of significant ecological and economic importance for capture fisheries and aquaculture in many parts of the world, with the flathead grey mullet being amongst the most important ones (Crosetti, 2016). Despite flathead grey mullets having a long tradition of being farmed in extensive and semi-intensive systems in earthen ponds or in cages, there exists very scarce information about the performance and nutritional requirements of these species in other types of aquaculture systems like RAS or aquaponic systems (Crosetti, 2016; Sadek, 2016). Current results showed that flathead grey mullet adapted well to both farming systems as indicated by survival and SGR when compared to other studies run in RAS (Bertini et al., 2023; Talukdar et al., 2020), whereas they performed better in relation to other reports (Garcés and Lara, 2023; García-Márquez et al., 2023), even though results are not directly comparable due to differences in the experimental conditions (i.e., water salinity and temperature, stage of development, and diet among others). Under current experimental conditions, when comparing both farming systems, flathead grey mullet reared in the aquaponic units performed better in terms of TGC than their congeners in the RAS. Two potentially complementary hypotheses might explain these results. In this sense, metabolites exuded by lettuce roots in aquaponic systems may influence water and fish gut bacterial communities, potentially enhancing fish growth (Ruiz et al., 2023). Additionally, water turbidity was significantly higher in aquaponic than in RAS tanks (data not shown), whereas this water increased turbidity might have benefited fish, since this species is well adapted to turbid environments (Whitfield et al., 2012), which may diminish fear responses (Leahy et al., 2011).

Regarding the production of *S. patula*, the overall glasswort yields were 47-55 g/m^2^/day, which were within the value range reported for *Salicornia ramosissima* species when using two different integrated multi-trophic aquaculture systems, based on the nutrient film technique (5-70 g/m^2^/day) and on deep water culture (25-320 g/m^2^/day) (Castilla-Gavilán et al., 2024). These results were also close to the values obtained in previous hydroponic studies (61 g/m^2^/day) (Kong and Zheng, 2014) and in a zero-water-exchange RAS-integrated multi-trophic aquaculture system (28 g/m^2^/day) (Waller et al., 2015). Nonetheless, under present conditions, the positive plant yield was partly due to the foliar fertilizer applied since the second month of trial to improve the plant yellowish and delayed growth. Yellowing is typically attributed to a deficiency in nutrients, causing a premature senescence and the accumulation of betalains, which are the pigments responsible for this particular coloration (Parida et al., 2018; Turcios et al., 2023). It is well demonstrated that the nutrient load in the medium is a pivotal factor in the growth of *Salicornia neei*, and that the lack of any nutrient negatively affects the plant yield (Diaz et al., 2020). Under this context, iron is a crucial micronutrient for the growth of plants in aquaponics, but its bioavailability is very limited (Kasozi et al., 2019). In addition, aquafeeds are low in iron due to its prooxidant characteristics as a transitional metal which can cause significant oxidative stress with potential damage to tissues and organs (Woodgate et al., 2022). Consequently, in coupled aquaponic systems where water is in constant recirculation, there may be a depletion of this mineral in the water. A similar consideration applies to other trace elements, such as nitrogen, phosphorus, and potassium (Graber and Junge, 2009; Tsoumalakou et al., 2023). However, this can vary between plant species and experimental set-ups; for instance, in our previous study we did not observe any sign of mineral deficiency in the different varieties of lettuce (Gisbert et al., 2024). In the present study, the application of the foliar fertilizer did not affect fish growth, as the growth performance of flathead grey mullets from the aquaponic system and RAS was very similar. These findings highlight the need for further research on aquafeed formulations specifically designed for growing fish in aquaponic systems, which ensure that fish and feed residues provide sufficient minerals and trace elements to promote proper plant growth.

### 4.2 Richness, diversity, and composition of the bacterial communities in the RAS and aquaponic systems

#### 4.2.1 Bacterial communities in the intestine of flathead grey mullet of the RAS and aquaponic systems

A high variability in estimated richness (Chao1 and ACE indices) and diversity (Shannon and Simpson indices) of the intestinal bacterial communities of flathead grey mullets reared in the aquaponic system (FIM2) was observed with respect to their congeners reared in the RAS (FIM1). Such variability may be attributed to the variations in oxygen and temperature water levels among aquaponics (lack of control of environmental water parameters). In this sense, a higher variability in fish bacterial richness and diversity is typically found in the gut of fish living in open environments with respect to those living in controlled aquaculture conditions (Kormas et al., 2014; Dehler et al., 2017; Viver et al., 2023). In addition, higher Shannon and Simpson diversity values were observed in the fish guts of the aquaponic system than in the RAS, which may respond to this adaptation to the environmental conditions. In this context, some studies in fish and higher vertebrates have correlated a high microbial diversity with an improved general condition and health, and a lower risk of gut colonization and infection by potential pathogens (Sekirov et al., 2010; Li et al., 2021). Thus, the increased gut microbial diversity observed in fish from the aquaponic system compared to those in the RAS might be a positive indicator of gut health, although this hypothesis cannot be confirmed without further research on the functional implications of such diversity increase.

The fish guts from both systems tested herein, aquaponic and RAS, were dominated by *Pseudomonadota* (FIM1: 89.1%; FIM2: 79.5%), in consistency with previous studies in flathead grey mullet reared in different environments (Le and Wang, 2020; Ofek et al., 2021; Bertini et al., 2023; Floris et al., 2024). The predominance of this phylum was mainly due to the high abundance of *Pseudomonas* (62.3-87.0%), which is one of the most dominant genera commonly observed in the intestine of marine fish (Egerton et al., 2018). The bacterial composition of the rest of bacterial taxa in the fish intestines varied between the RAS and the aquaponic systems, in agreement with the differences in inter-individual phylogenetic diversity (beta diversity based on weighted UniFrac distances). For instance, the relative abundance of *Bacillota* was higher in FIM1 than in FIM2, in line with the higher abundance of *Paenibacillus* (Δ = −5.9%). Strains belonging to the genus *Paenibacillus* have been demonstrated to have probiotic effects in fish, improving their growth and feed performance, antioxidant activities, immune response, intestinal health, and disease resistance against potential pathogens (Amoah et al., 2021; Midhun et al., 2019). In FIM2, there was a higher relative abundance of the phylum *Planctomycetota*, in relation to the higher levels of the genera *Blastopirellula* (Δ = + 1.4%) and *Pirellula*-related Pir4 lineage (Δ = + 1.0%), and phylum *Actinomycetota*, mainly due to the higher abundance of *Mycobacterium* (Δ = + 1.8%). Overall, all the above-mentioned genera have been previously described as part of the commensal gut microbiota of teleost fish (Dawood, 2021; Yu et al., 2021; Luo et al., 2022; Najafpour et al., 2023; Ruiz et al., 2023), which combined with the successful results of fish growth indicates that both rearing systems provide a good environment for ensuring a favorable fish growth and intestinal health. In addition, some genera such as *Vibrio, Aeromonas*, and *Flavobacterium*, which are a major burden in aquaculture systems since they have been more frequently recognized as potential fish pathogens (Banerjee and Ray, 2017; De Silva and Heo, 2023), were absent or showed very low abundances in the fish gut and across the compartments of both RAS and aquaponic system.

#### 4.2.2 Bacterial communities in the water of the RAS and aquaponic systems

The water showed a progressive increase in bacterial richness (Chao1 and ACE indices) and diversity (Shannon and Simpson indices) throughout the RAS unit (fish tank, WFT1; sand filter, WSF1; and biological filter, WBF1), indicating that the water may act as substrate and as a medium of transport for the bacteria within the system, as previously reported in other studies (Khalil et al., 2021; Dahle et al., 2022). In this sense, communities found in the water flow of an aquaculture system typically include groups of bacteria originated in the source water, detached from biofilms, and bacteria coming from the microbiota of the farmed species (Moschos et al., 2022). In contrast to the results of RAS, no differences in intra-individual richness and diversity, nor on inter-individual diversity in terms of weighted UniFrac distances were found among the water from the different compartments of the aquaponic systems (sedimentation tank, WST2; hydroponic tank, WHT2). The origin of the different dynamics between both systems may be that the IRTAmar^®^ RAS is equipped with an UV lamp just after the biological filter, so the diminishment in bacterial richness and diversity from WBF1 to WFT1 could indicate its effectiveness in reducing the bacteria of the water in recirculation (Dahle et al., 2022), whereas water microbiome richness and diversity were more stable along the hydraulic circuit within the coupled aquaponic unit (Ruiz et al., 2023).

*Pseudomonadota* and *Bacteroidota* were the predominant phyla in the water from both rearing systems, which are typically the most predominant in the water of recirculation systems (Duarte et al., 2019; Moschos et al., 2022; Ruiz et al., 2023). Members of the phylum *Pseudomonadota* are highly versatile in their metabolic capabilities, playing important roles in nutrient cycling and the mineralization of organic matter. In contrast, *Bacteroidota* are heterotrophic bacteria primarily involved in cycling of substances rich in complex carbons and proteins (Moschos et al., 2022), and their higher abundance in the coupled aquaponic system compared to the RAS may be attributed to the higher availability of total available carbon, the mineralization of solid waste and higher levels of dissolved oxygen typical of aquaponic units (Kasozi et al., 2021). Thus, the lower abundance of *Pseudomonadota* in the water of the aquaponic system with respect to the RAS may be attributed to the absence of many genera belonging to this phylum such as *Leucothrix, Pseudomonas, Pseudoalteromonas*, and *Litoreibacter*. The changes in water composition between both systems may also in part be due to the different bacterial richness and diversity in the water circulating across the different compartments between both systems. Reinforcing this hypothesis, the differences in the abundance of many bacterial genera between the water from both systems were correlated with differential abundances in the other analyzed habitats from both systems. For instance, the absence of *Leucothrix* in the water of the aquaponic systems can also be linked to its absence in the biological filter and biofilms growing in the walls of the hydroponic tanks, whereas it was present at high abundances in the three types of habitats within the RAS unit (water, WFT1, WSF1 and WBF1: 3.5-17.0%; bioballs, BBF1: 6.4%; biofilms, BFT1, BET1 and BDC1: 14.57-17.88%). In this sense, the water of marine or brackish RAS is characterized by the predominance of aerobic chemoorganoheterotrophic genera, including *Leucothrix* (Moschos et al., 2022). Moreover, the addition of iron as a foliar fertilizer in the aquaponic system would also have altered the chemistry of the seawater in the tanks, as seen in open ocean field experiments in iron-depleted waters where attempts have been made to boost phytoplankton production as a means of carbon sequestration (Turner et al., 2004). The additional consequence of the iron supplementation in these open ocean experiments was the transformation of some sulfur compounds to gaseous forms of sulfur (dimethylsulfide) that were released to the atmosphere. Thus, in the context of the aquaponic system, this supplement may have reduced available sulfur required by some members of the bacterial genus *Leucothrix*, whose members include sulfur-dependent chemo lithoautotrophs (Teske and Salman, 2014; Riedel et al., 2013; Cuhel et al., 1981).

Concerning the absence of *Litoreibacter* in the water of the aquaponic system (WST2, WHT2 and BHT2), it was correlated with its absence in the biofilm of the hydroponic tank (BHT2), while it was present in the RAS water from the fish tanks (WFT1, 5.5%) and from the biological filter (WBF1, 2.9%), as well as in the biofilms from the RAS unit (BFT1, BET1 and BDC1, 4.4-11.5%). This bacterial genus from the *Rhodobacteraceae* family whose members are phototrophs and chemotrophs, has been previously isolated from the water of marine aquaculture sites (Kanamuro et al., 2021) and of saltwater RAS units (Bastos Almeida et al., 2022). On the other hand, the presence of *Mycobacterium* in the water of the aquaponic unit (1.1-4.2%) may be related to the abundance of this genus in the fish intestines (1.8%), bioballs (4.4%) and biofilm from the walls of the hydroponic tank (4.6%), while it was absent in the different habitats of the RAS. Overall, these results underscore the importance of the water as a dynamic conduit for bacterial dispersal, facilitating the exchange of microbes from biofilms and the microbiota of cultured fish species across interconnected compartments, which was especially evinced when examining bacterial diversity and composition in the biofilms of each system.

#### 4.2.3 Bacterial communities in the biofilms of the RAS and aquaponic systems

As occurred with the water, there was an increase in bacterial richness (Chao1 and ACE indices) and diversity (Shannon and Simpson indices) throughout the biofilms from the RAS. The decrease in bacterial richness and diversity in the biofilm from the walls of the degassing column (BDC1) with respect to BFT1 and BET1 may respond to the reduction in richness and diversity of the bacterial communities of the water after the UV filter, and likely to the effect of the sand filter as a physical barrier to retain suspended solids which containing biofilm-forming bacteria adhered on their surface (Dey and Raghuwanshi, 2021). On the other hand, the bacterial ACE richness and diversity (Shannon and Simpson indices) of the biofilms collected from the aquaponic system (walls of the hydroponic tanks, BHT2; roots of glasswort, BRG2) showed similar values between them, in line with the constant values of bacterial richness and diversity in the water.

The differences in microbial community structure (weighted UniFrac distances) between both systems were explained by their different taxonomic composition. On one hand, the biofilms collected from the RAS were dominated by the phyla *Pseudomonadota* (60.0-79.7%) and *Bacteroidota* (11.0-24.3%). On the other hand, the biofilm from the walls of the hydroponic tanks showed high predominance of *Planctomycetota* (35.3%), *Pseudomonadota* (34.3%) and *Actinomycetota* (10.4%), and the biofilms from the roots of glasswort were dominated by *Pseudomonadota* (34.2%), *Planctomycetota* (25.1%), and *Actinomycetota* (14.6%). At the level of genus, the biofilms from BHT2 and BRG2 were dominated by bacterial genera like *Fuerstiella* (9.1% and 3.4%, respectively), *Blastopirellula* (6.6% and 8.0%), *Mycobacterium* (4.6% and 7.9%) and *Hoeflea* (4.4% and 0.9%), and *Marivita* (0.44% and 1.4%) that were also present at significant abundances in the water of the aquaponic system, and near absent in the biofilms of the RAS. Previous studies have reported the presence of the heterotrophic genera *Fuerstiella* and *Blastopirellula* in the biofilm adherent to the biofilters of seawater RAS (Hüpeden et al., 2020; Kushwaha et al., 2023), and *Blastopirellula* in the biofilms of concrete and plastic surfaces in marine environments (Wang et al., 2021; Karačić et al., 2022). *Mycobacterium* has been reported forming biofilms in dry and mineralized zones and in the plant roots of a flood-and-drain media bed aquaponic system (Kasozi et al., 2020), in line with the results of the present trial. Some members of *Mycobacterium* have plant growth-promoting properties, including siderophore production, ammonia production and phosphate solubilization, which brings focus to the importance of the presence of these bacteria in plant roots within agriculture systems (Sanchez et al., 2019). In addition, the species *Hoeflea suaedae* has also been isolated from the roots of the halophyte *Suaeda maritima* (Chung et al., 2013)-which is in fact a likely synonym for *S. patula*, the species studied herein (Loidi et al., 1999). Moreover, *Hoeflea* have been isolated from iron-containing sediments since they can oxidize iron (Sorokina et al., 2012) so its presence, as well as the optimal growth of the glasswort, may be indicators of a sufficient iron availability in the aquaponic system after the application of the foliar fertilizer. Further, some strains belonging to the genus *Hoeflea* have been characterized as putative producers of bioactive compounds, including bacteriocins, polyketides, and auxins (Bentzon-Tilia et al., 2014), which have beneficial properties that ensure food safety and plant growth, and aid in control of plant diseases (Teale et al., 2006; Han et al., 2018; Lahiri et al., 2022). In addition, the genus *Marivita* plays a significant role in sulfur and carbon biogeochemical processes, as well as in symbiotic relationships with aquatic microorganisms (Addo et al., 2021).

The biofilms collected from the RAS showed high abundance of genera nearly absent in the biofilms of the aquaponic system and which were also present at significant abundances in the water of the RAS, such as *Leucothrix* (up to 17.9%), *Litoreibacter* (up to 11.5%), *Halomonas* (up to 9.1%), *Muricauda* (up to 6.0%), and *Maribacter* (4.2%). Many of these genera have been previously reported in the water or the biological filter of marine RAS (Lu et al., 2019; Diéguez et al., 2020; Moschos et al., 2022; Zhang et al., 2023). Some of these microorganisms may play a role in chemical oxygen demand and biotransformation of organic carbon within the biofilms, such as *Maribacter* which participates in the decomposition of a variety of organic compounds (Gao et al., 2012). Regarding *Sulfitobacter*, which was present on the different biofilms of both systems, it is a heterotrophic genus which has been found as dominant in the microbial communities associated to the water and biofilm adherent within the biofilter of marine RAS (Gao et al., 2012; Duarte et al., 2019; Diéguez et al., 2020). Some members belonging to this genus have shown inhibitory activity toward potential fish pathogens, like *Vibrio anguillarum* (Sharifah and Eguchi, 2012), potentially supressing their development in these systems (Duarte et al., 2019). In addition, strains of *Sulfitobacter* have a role in sulfur cycling within these systems due to their ability to oxidize sulfite (Bourne et al., 2004; Xu et al., 2024), which explain its greater abundance in more aerobic habitats like the water with respect to the biofilms.

Overall, the predominant genera found in the biofilms associated to biotic and abiotic surfaces from both systems were aerobic and chemo organoheterotrophic, which indicates that their growth may result from the presence of organic compounds derived from feed and fish feces in the water (Bartelme et al., 2019; Kasozi et al., 2020). However, contrary to the results of water, all the biofilms, including those from the different compartments within the RAS, showed a differential inter-individual phylogenetic diversity among them, as represented in the PCoA. These differences may stem in part from different selection pressures across compartments, including differences in oxygen levels, organic matter accumulation (Fischer, 2003), nutrient availability (Diaz et al., 2023) and variations in hydraulic retention times (Nogueira et al., 2002) or flow rates (Khu et al., 2023). This was reflected not only in the above-mentioned dissimilar bacterial composition among systems, but also on the specificity in the composition from each compartment within the RAS. In particular, the bacterial genus *Sphingorhabdus* and *Maribacter* were found at high abundance in BFT1 (6.2% and 4.2%, respectively) and almost absent in the rest of the biofilms from the RAS, while *Halomonas* and *Nautella* were only observed in BET1 (at 9.1 and 7.1% abundances, respectively). In BDC1, *Muricauda* (6.0%), *Sulfitobacter* (4.2%) and *Pirellula*-related Pir4 lineage (3.5%) were the only genera which were present at an abundance higher than 1%, even though they were also present in BET1 at lower abundances. In line with these findings, previous studies have reported a progressive growth of certain heterotrophic bacteria after UV irradiation since it increases nutrient availability and reduces resource competition, favoring their colonization (Hess-Erga et al., 2010; Dahle et al., 2022). Further, the mechanism of action of UV irradiation is based on the genotoxic DNA damage caused on the microorganisms present in the water, leading to mortality (Schuch and Menck, 2010). However, the effectiveness of the UV filter in a RAS is dependent on the species-specific tolerances to UV light, as well as on the hydraulic retention times, the turbidity of the water, and the particles in the water, which can lead to “fouling” of UV systems (Dahle et al., 2022). In the present study, the fact that *Leucothrix* and *Litoreibacter*, which showed high abundances in BFT1 and BET1, were very close to 0% in BDC1 after the UV filter, indicates the efficacy of the UV filter and of the previous physical filtration systems in desinfection. Thus, a more plausible explanation for the persistence of *Muricauda, Sulfitobacter* and *Pirellula*-related Pir4 lineage may be related to the use of specific DNA repairing mechanisms after the UV irradiation (Schuch and Menck, 2010). For instance, the members of the phylum *Planctomycetota* have shown high resistance to UV irradiation through protective DNA damage repair mechanisms, especially in the strains phylogenetically similar to *Rhodopirellula baltica*, and *Pirellula*-related Pir4 lineage (Storesund and Øvreås, 2013; Viana et al., 2013). In addition, some species of *Muricauda* have also shown UV resistance, which has been attributed to their carotenoid content (Núñez-Pons et al., 2018); findings which are also consistent with the results of the present trial.

#### 4.2.4 Bacterial communities adhered to the biological filters of the RAS and aquaponic systems

Regarding the bacterial communities adherent to the biological filter, there were no differences in richness (Chao1 and ACE) and diversity (Shannon and Simpson) of both systems. In the present study, the use of the bioballs as carriers led to significant abundances of the genus *Nitrosomonas* in the biofilter of both systems (BBF1: 3.8%; BBF2: 1.8%). This genus, typically found in biological filters, consists of nitrifying bacteria responsible for the elimination of toxic nitrogen compounds, like the ammonia excreted by fish, through their oxidation into nitrate (Schreier et al., 2010). Apart from containing nitrifying bacteria, the bacterial communities primarily found in biological filters also consist of heterotrophic denitrifying bacteria, which remove the carbon and nitrate accumulated from uneaten feed and fecal matter, as is the case of *Pseudomonas* and *Blastopirellula* (Schreier et al., 2010; Hüpeden et al., 2020), which were also found in the bioballs of both systems in the present study (BBF1: 2.0% and 0.2%; BBF2: 0.4% and 8.2%, respectively). The results agreed with numerous studies on the composition of the biofilm attached to the carriers from the biological filter of recirculating systems. For instance, Guo et al. (2025) reported significant abundances of *Bythopirellula, Blastopirellula, Nitrosomonas, Fuerstiella, Planctomicrobium, Rubritalea* and *Rhodopirellula*, in the carriers of a RAS for rearing turbot (*Scophthalmus maximus*), while Almeida et al. (2021) reported *Blastopirellula, Leucothrix, Nitrosomonas*, and *Rubritalea* in a RAS for growing sole (*Solea senegalensis*). Among them, *Leucothrix*, which was present at high abundance in BBF1 (6.4%), has been positively correlated with the concentration of nitrate in RAS biofilters (Almeida et al., 2021), indicating its potential to create consortia with nitrifying bacteria. On the other hand, *Rhodopirellula* is a genus of chemoheterotrophic aerobic marine bacteria, and the species *Rhodopirellula baltica* has been shown to live freely under ammonia limiting conditions, but it forms biofilms in presence of ammonia (Frank et al., 2011). In line with this, the presence of this genus in the biofilms associated to the bioballs (0.2% in BBF1, and 2.0% in BBF2) may indicate that its members may be able to dwell together with denitrifying bacteria. Such functionally diverse biofilms reflect an enhanced nitrogen removal efficiency and contribute to water quality stability in recirculating systems (Schreier et al., 2010; Rurangwa and Verdegem, 2015). Regarding the different abundances of bacteria observed generally associated to the analyzed habitats, they may be attributed to the differences in the design of each system, particularly to the different environmental conditions (controlled conditions *vs*. ambient conditions), to the use of UV water filtration in RAS, to the effects of the metabolites exuded in the water by the roots, and to the effects of iron supplementation (foliar fertilizer), among others. Overall, the presence in the biofilters of the RAS and aquaponic system of both nitrifying and denitrifying bacteria, and other bacteria able to cohabit under the conditions generated by them, reflects an equilibrium in the bacterial composition of the biofilms adherent to the biofilter and evinces the functional effectiveness of the biofilter in each system, which highlights their application for microbial-based water treatment in aquaculture. In this context, despite the greater variability observed in the aquaponic system due to less regulated conditions aimed at reducing operational costs (Supplementary Figure 1), ammonia, nitrite, and nitrate levels remained within the commonly reported non-toxic ranges for comparable aquaculture systems (0.1-2.9 mg ${\text{NH}}_{4}^{+}$/L, 0.4-4.4 mg ${\text{NO}}_{2}^{-}$/L, and 10-200 mg ${\text{NO}}_{3}^{-}$/L; Duarte et al., 2019; Wongkiew et al., 2017; Tumová et al., 2025), including the RAS (Supplementary Figure 2). The balanced concentrations of these nitrogenous compounds also align with satisfactory fish growth and survival in both systems, underscoring the importance of nitrifying and denitrifying bacteria in system performance. Moreover, although this trial involved a relatively low initial stocking density, the establishment of microbial communities involved in nitrogen cycling suggests that both RAS and aquaponic systems could support higher fish stocking densities without compromising water quality. Nevertheless, further research would be required to determine the optimal stocking density for each system in terms of water quality management and fish and plant growth and welfare. In this regard, efficient microbial regulation of ammonia and nitrite levels is a key prerequisite for system intensification in recirculating aquaculture (Martins et al., 2010; Schreier et al., 2010).

## 5 Conclusions

This study demonstrates the good adaptability of flathead grey mullet to grow in an indoor RAS under controlled conditions and a coupled aquaponic system using marine water under ambient conditions within a greenhouse (early spring-end of summer), which paves the way toward the use of this species for sustainable aquaculture practices. Moreover, both rearing systems provided a good environment for ensuring the fish intestinal health, as suggested by the high abundance of the probiotic bacterial genus *Paenibacillus* in the gut of the fish from the RAS unit, along with other genera observed for both rearing systems which are part of the typical commensal microbiota of this species. In addition, the results also proved the effectiveness of the UV filtration from the RAS unit in reducing the bacterial richness and diversity in the water, which was also observed in the biofilms, evincing the role of the water as a transport for the bacteria between compartments. The presence of some bacterial members in the water was attributed to their high abundance in other habitats from the systems, such as biofilms from abiotic surfaces, from the fish gut and from plant roots. For instance, *Mycobacterium, Sulfitobacter* and *Marivita* were found in both the water of the aquaponic system and the roots of glasswort. These bacterial genera may also have a role in supporting plant growth and health by potentially suppressing fish pathogens and promoting nutrient cycling for the benefit of the plants. On the other hand, the microbial communities of the biofilms from the RAS unit were very different to the aquaponic system, with high-abundant genera such as *Leucothrix* and *Litoreibacter*, which have already been reported in marine or brackish RAS. The differential abundances found in the bacterial composition between the RAS and aquaponic system were likely due to the distinct environmental conditions and component design of each system. In addition, the biofilm composition among compartments was very different within the RAS unit, reflecting how the operation and design of each compartment create distinct environmental conditions that shape the resident microbial communities. Furthermore, the bacterial communities found on bioballs of the biological filters of both systems were in line with previous results of the microbiome typically associated to carriers in biological filters for these types of systems. The presence of the ammonia-oxidizing bacterial genus, *Nitrosomonas*, and of the heterotrophic denitrifying genera, *Pseudomonas* and *Blastopirellula*, serves as an indicator of the biofilters' capacity to efficiently convert nitrogenous waste products and supports their role in both RAS and aquaponic systems, from a food security perspective.

## Data Availability

The data presented in this study are publicly available. The data can be found here: https://www.ncbi.nlm.nih.gov/bioproject/PRJNA1309991.
