# Supplementary material for: System design and habitat type drive microbial communities in recirculating aquaculture systems: comparison of conventional fish-only and sustainable aquaponic systems
Source: Front Microbiol. 2026 Feb 24;17:1706522. doi: 10.3389/fmicb.2026.1706522 (PMC12971695; doi:10.3389/fmicb.2026.1706522)
Supplement: Supplementary file 1 [file Supplementary_file_1.docx]

Supplementary Material


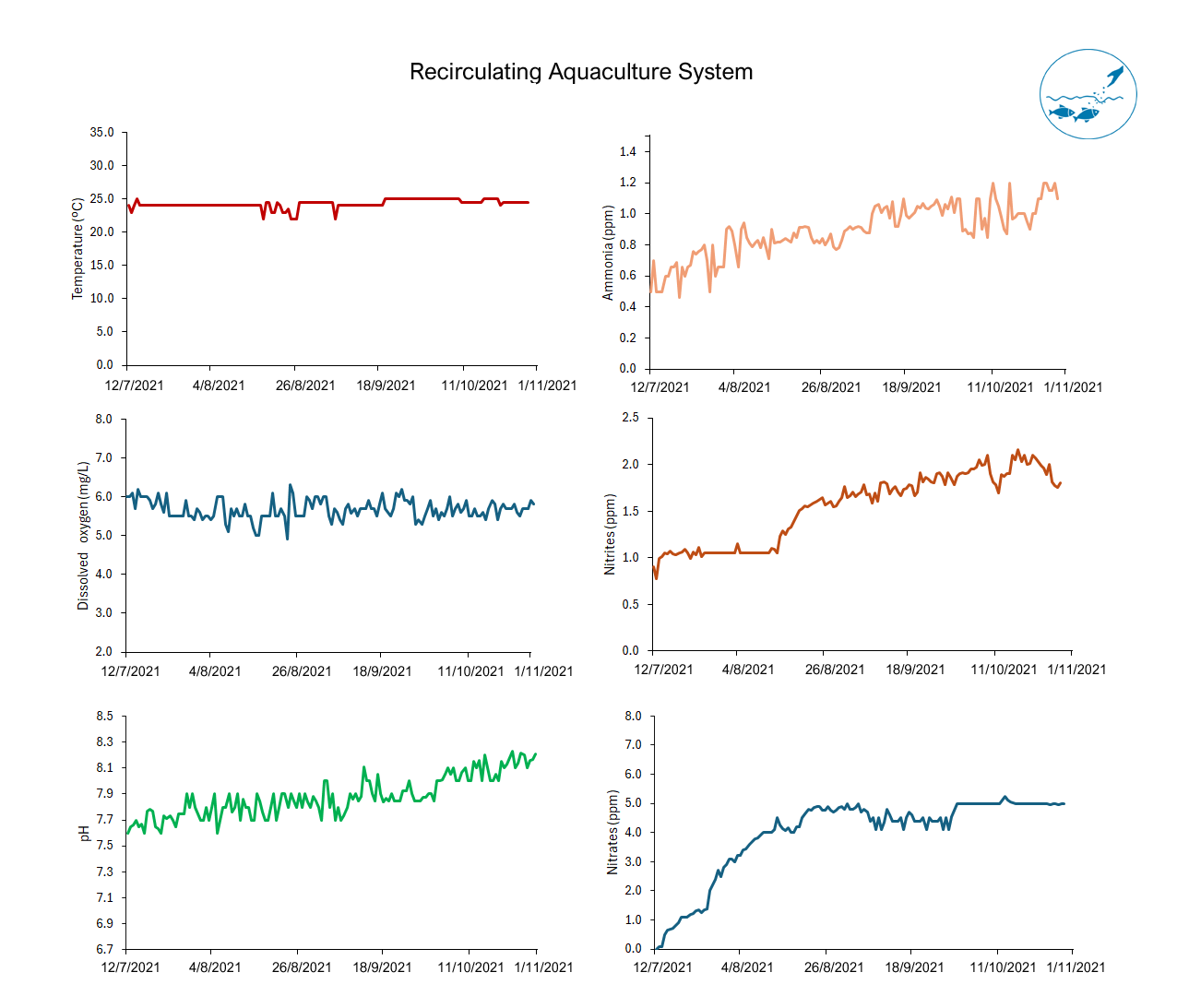


**Supplementary Figure 1.** Recorded values of temperature, dissolved oxygen, pH, ammonia, nitrite, and nitrate levels in the water of the tanks of flathead grey mullet (*Mugil cephalus*) from the recirculating aquaculture system (12^th^ July to 1^st^ November 2021).


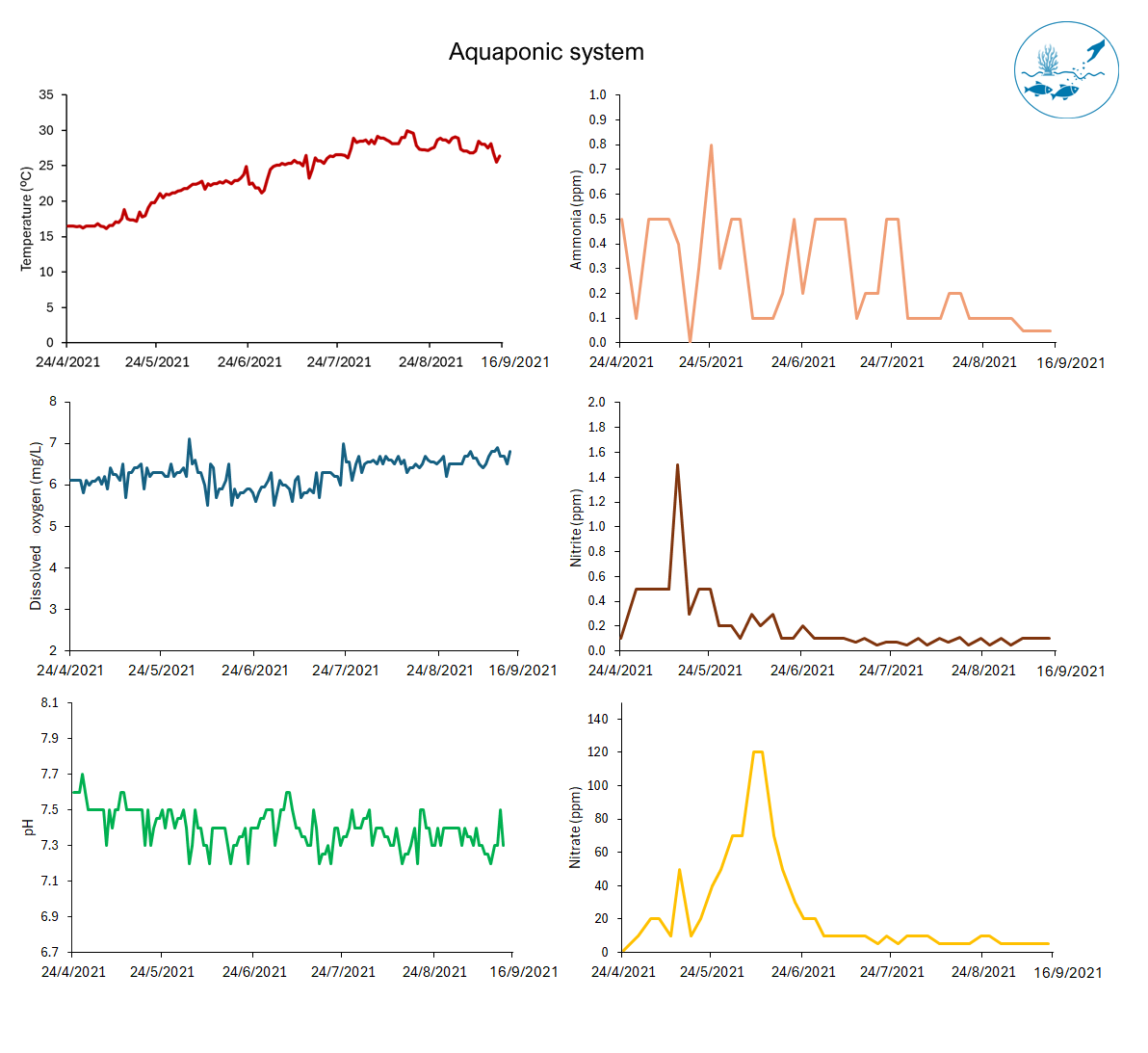


**Supplementary Figure 2.** Recorded values of temperature, dissolved oxygen, pH, ammonia, nitrite, and nitrate levels in the water of flathead grey mullet (*Mugil cephalus*) tanks from the aquaponic system during the experimental period (24th April to 16th September 2021).
